# Innovative integration: optimizing performance through warm-up and photobiomodulation in high-intensity test

**DOI:** 10.3389/fspor.2024.1341106

**Published:** 2024-01-18

**Authors:** Izabela A. Santos, Marina Paiva Lemos, Enrico Fuini Puggina, Gustavo R. Mota

**Affiliations:** ^1^ Graduate Program in Rehabilitation and Functional Performance, Ribeirao Preto Medical School, University of São Paulo (USP), Ribeirão Preto, Brazil; ^2^ Exercise Physiology in Health and Human Performance Research Group, Department of Physical Education, University of Uberaba (UNIUBE), Uberaba, Brazil; ^3^ Exercise Science, Health and Human Performance Research Group, Department of Sport Sciences, Institute of Health Sciences, Federal University of Triangulo Mineiro (UFTM), Uberaba, Brazil

**Keywords:** laser therapy, team sports, ergogenic aids, running, female, futsal, soccer (football), performance

## Abstract

We investigated whether the application of photobiomodulation therapy (PBMT) immediately after a standardized warm-up (WU + PBMT) or traditional PBMT (no pre-warming) would influence performance in intermittent testing and intensity variables. In a counterbalanced randomized crossover design, twelve female futsal players (mean age: 23.9 ± 3.8 years) attended four sessions. Each session involved either a standardized warm-up or maintaining seated rest for five minutes. Subsequently, PBMT or placebo (with the PBMT device turned off) was applied, followed by the YoYo Intermittent Recovery Level 1 test (YYIR1) during which we assessed heart rate, rating of perceived exertion, and blood lactate levels. The performance in YYIIR1 was superior (*p* = 0.02) in the WU + PBMT condition (440.0 ± 59.0 m) compared to the WU + Placebo (353.3 ± 94.7 m), and placebo alone (no warm-up) (325.0 ± 67.2 m). We conclude that a combination of a specific warm-up before PBMT application improves high-intensity intermittent performance in amateur female futsal players without affecting intensity variables.

## 1 Introduction

A futsal match demands intermittent and high-intensity efforts, along with brief recovery periods, imposing physiological demands on both aerobic and anaerobic pathways, notably the phosphagen system (1). In response to these requirements, the potential positive effects of photobiomodulation therapy (PBMT) on mitochondrial enzymatic processes for ATP production (2) suggest a pathway toward enhanced phosphocreatine re-synthesis, offering opportunities for optimizing performance (3) PBMT, also known as low-level laser therapy, has surfaced in sports as a potential non-pharmacological ergogenic aid for enhancing physical performance and recovery (4, 5). PBMT primarily operates at the muscular and metabolic levels, stimulating adenosine triphosphate (ATP) production through activation of mitochondrial complex IV. This activation enhances electron flow in the respiratory chain, leading to an increased quantity of H^+^ ions (6). Additionally, PBMT stimulates satellite cells, augmenting muscle regeneration capacity (7).

In practical terms, these effects are translated as improvement of running performance. For example, endurance runners (VO_2max_ of 63.2 ± 6.4 ml·kg^−1^·min^−1^) performed a 3 km running test ∼7 s faster after PBMT when compared to placebo. Additionally, pre- and post-exercise PBMT applications promoted changes in VO_2_ response (PBMT = 40.3 ± 4.2 vs. Placebo = 42.3 ± 4.4 ml.kg^−1^.min^−1^) evaluated at a specific speed (12 km·h^−1^), improving the metabolic economy of runners 24 h after the 3 km test (8).

Warm-up is a well-established non-pharmacological strategy for ergogenic purposes in sports. Despite widespread use by coaches and trainers, scientific exploration of warm-up effects on sports performance is relatively recent (9). Various mechanisms contribute to warm-up effects, including elevated body temperature (10), enhanced muscle metabolism (ATP resynthesis), improved muscle fiber performance (heightened ATP and phosphocreatine levels) (11), optimized oxygen uptake kinetics (12), and impacts on neural, mechanical, and psychological aspects (13).

Despite supporting warm-up as an ergogenic resource, discussions in the literature persist. In the context of futsal, the impact of different durations and the exploration of warm-up routines in elite teams have been examined elsewhere (14, 15). Notably, in team sports, specific warm-ups demonstrate greater effectiveness compared to generic ones (16). The YoYo Intermittent Recovery test Level 1 (YYIR1) stands out as a suitable specific activity for elevating body temperature in high-intensity intermittent team sports, owing to its established connection between the test and match performance (17). Additionally, studies in futsal players have indicated that YYIR1 performance was ∼20% better in professional seniors than in younger under-20 players (18).

Warm-up and PBMT are recognized performance-altering strategies, positively impacting muscular and metabolic aspects (4, 9). However, the combined effects of these two strategies remain unknown. Krustup et al. (2015) (19) identified a significant correlation between maximal oxygen uptake, muscle citrate synthase activity, and distance covered in high-intensity intermittent tests, specifically for untrained individuals. This correlation was not observed in trained individuals, suggesting a more prominent role of aerobic energy production in fatigue resistance for untrained subjects during intense intermittent exercise (19). Considering PBMT's potential to enhance mitochondrial membrane potential and ATP synthesis in C2C12 myotubes (20), as well as its demonstrated ability to increase oxygen availability in healthy young participants (21), investigating the effects of PBMT alone or preceded by a warm-up on YYIR1 performance in amateur players would be intriguing.

Therefore, we evaluated whether the application of PBMT immediately after a standardized warm-up (WU + PBMT) or the traditional PBMT (no warm-up before) would influence maximal intermittent exercise performance and physiological indicators in amateur female futsal players. We hypothesized that the combination of warm-up and PBMT would improve high-intensity intermittent performance.

## 2 Methods

### 2.1 Subjects and ethical care

Twelve female amateur futsal players regular participants in state and national-level amateur championships (mean age: 23.9 ± 3.8 years; anthropometrics: 63.2 ± 8.3 kg, 1.61 ± 0.3 cm, 27.7 ± 4.5% body fat; futsal experience: 12.9 ± 4.5 years; 3 h weekly training) participated without any contraindicating injury history. Local Ethics Committee approval was obtained under process number 4.493.200/2021. Sample size calculation, based on a PBMT sports study (22) (effect size: 0.8; test power: 0.8), with β at 20% and α at 5%, mandated a minimum *n* = 10 for discerning PBMT vs. placebo effects.

### 2.2 Study design

**Figure 1:**
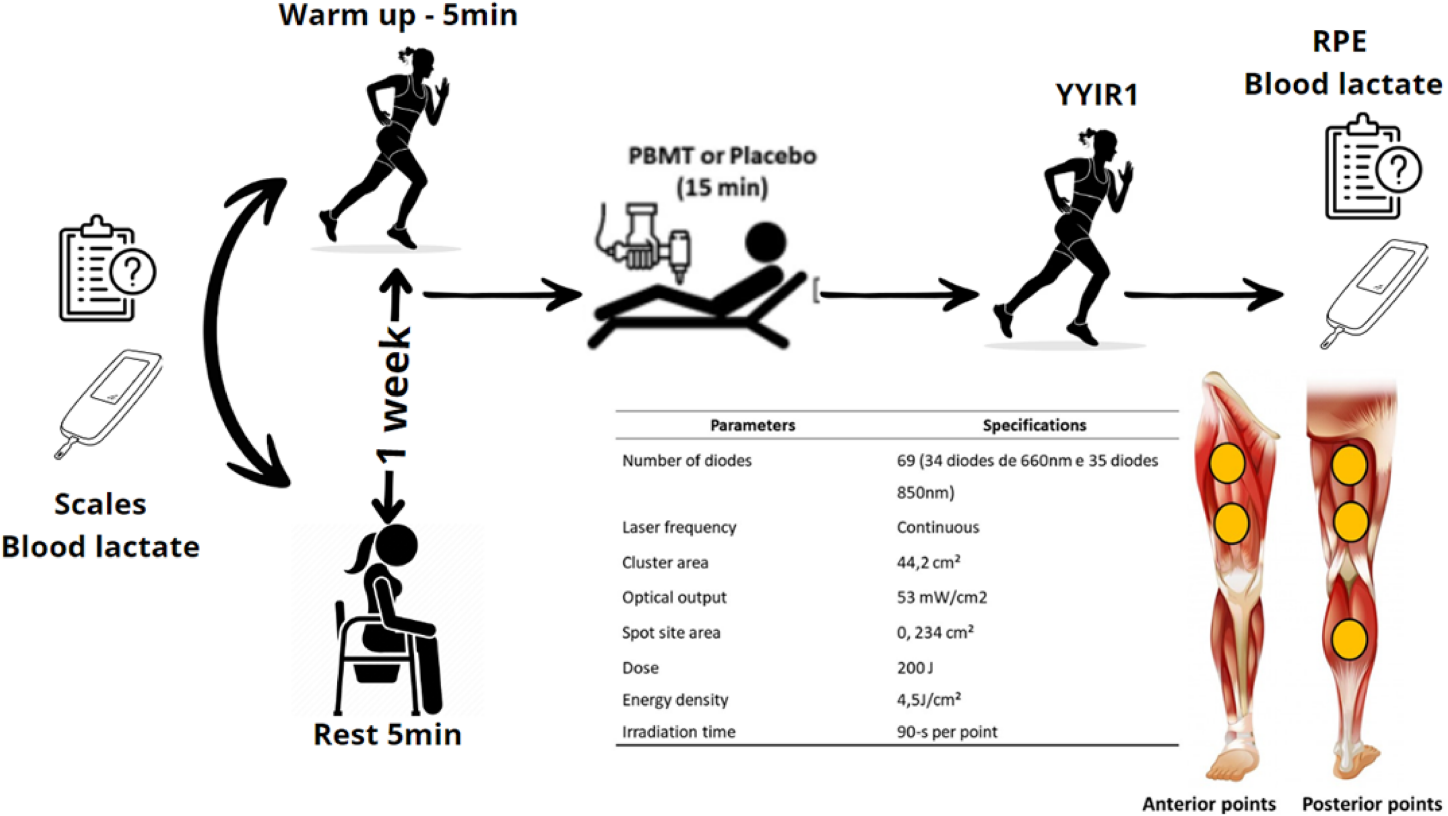
General experimental design (*n* = 12) and details of photobiomodulation therapy (PBMT) parameters; YoYo intermittent recovery test level 1 (YYIR1). Heart rate (HR) recorded during and after tests; rating of perceived exertion (RPE). The participant remained blinded to the protocols (PBMT or placebo), the PBMT applicator to YYIRT1 execution, and the YYIRT1 tester to the recent protocol.

The study employed a blinded, randomized, placebo-controlled crossover design, with 12 participants undergoing four distinct conditions (one-week intervals). Given PBMT effects up to 48 h post-application (23), the chosen interval prevented residual effects. A total of 48 (i.e., 12 × 4) experimental observations were obtained. Prior to each session, participants underwent two familiarization sessions for the YYIR1 test and the utilized perceptual scales. See Figure 1 for experimental procedures.

Participants trained for 3 h weekly (1 h each on Monday, Wednesday, and Friday). Experimental sessions took place on Mondays during the pre-competitive phase. Conducted by the same researcher in a controlled environment (27°C ∼50% relative humidity) at consistent times, tests took place during four individual sessions, spaced one week apart. Upon arrival, players reported perceived recovery status (PRS) and delayed onset muscle soreness (DOMS) using visual scales. After collecting a 25 *µ*l blood sample for lactate concentration, we applied PBMT or placebo (device turned off) immediately following a standardized warm-up or five-minute rest. The YYIR1 test followed, with recording of perceived exertion (RPE) and blood lactate. Heart rate (HR) was monitored during both warm-up and the YYIRT1.

Data collection involved independent researchers. The individual administering PBMT or placebo was blinded to test execution details to ensure impartiality. Participants were instructed to abstain from alcohol and substances that could affect performance. Strenuous exercise was to be avoided 48 h before each session in all study conditions.

### 2.3 Perceived recovery status (PRS) and muscle soreness (DOMS) status

Upon laboratory arrival, players reported their PRS, graded in arbitrary units (AU) from 0 to 10. A score of 0 indicated “very poorly recovered/extremely tired,” while 10 denoted “very well recovered/very energetic” (24). DOMS was assessed via a visual scale ranging from 0 to 10, with 0 indicating “no pain” and 10 signifying “maximum pain” (25), ensuring uniformity across experimental conditions.

### 2.4 Blood lactate concentration, heart rate (HR) and rating of perceived exertion (RPE)

Blood lactate concentration (∼25 *µ*l from the finger) was assessed pre- and post-YYIR1. HR during warm-up and YYIR1 was monitored using the Polar Team System™ (Kempele, Finland), recording minimum, average, and peak HR values. Post-warm-up and YYIR1, RPE was reported via the CR-10 Borg scale (26).

### 2.5 Standardized warm-up

The standardized warm-up involved three repetitions of YYIR1 levels 1–3. Upon completing level 3, the test audio automatically reverted to level 1, repeated thrice. This warm-up, lasting ∼5 min, aimed for specificity to both YYIR1 and futsal matches (17, 18). Pilot study tests revealed a ∼76% HRmax response.

### 2.6 Photobiomodulation therapy (PBMT) protocol

Following warm-up or a 5-minute rest, PBMT (or placebo) application commenced using a THOR™ Photomedicine red and infrared LED device (London, UK). Application time per point, calculated for precise energy delivery (200 J), was 1 min and 30 s, with 5 standardized points on each limb: two in quadriceps, two in hamstrings, and one in the gastrocnemius. The total application duration was 15 min (27), with point selection based on their involvement in running exercises like YYIR1 (28).

In both PBMT and SHAM conditions, players remained unaware (blinded) of treatment due to eye covering and ear mufflers, preventing visual and auditory cues during irradiation. Placebo procedures mirrored those of PBMT (points and time), but the PBMT equipment was inactive (27). The detailed parameters of PBMT are specified in Figure 1.

### 2.7 Yoyo intermittent recovery test level 1 (YYIR1)

The YYIR1, a cost-effective and reproducible test, yields crucial data on physical conditioning and physiological parameters for females (29). It involves intermittent 2 × 20 m runs with progressively increasing speeds (starting at 10 km·h^−1^), guided by specific audio cues (10 s recovery in a 5 m demarcated area). Test termination occurs if the player fails to maintain pace or voluntarily withdraws. Verbal stimuli were standardized, and the testing researcher remained unaware of the applied intervention (30).

### 2.8 Statistical analysis

The Shapiro-Wilk test assessed data distribution. Paired T tests compared warm-up conditions, and one-way ANOVA with Tukey's *post hoc* analyzed the four conditions (WU + Placebo / WU + PBMT / placebo / PBMT). Significance was set at 0.05. Effect size (ES) for YYIR1 performance determined differences' significance, classified as trivial (<0.2), small (>0.2–0.6), moderate (>0.6–1.2), large (>1.2–2.0), and very large (>2.0) (31).

## 3 Results

Regarding warm-up, HR values and RPE did not differ among conditions (*p* < 0.05). Table 1 presents variables measured during the 5 min before the YYIR1.

**Table 1:** Heart rate (HR) and rating of perceived exertion responses (RPE) during the 5 min before the YoYo intermittent test level 1 (YYIR1).

	PBMT	Placebo	WU + PBMT	WU + Placebo
HR minimum (bpm)	NA	NA	101.6 ± 13.5	103.7 ± 12.6
HR mean (bpm)	70.8 ± 2.4	70.7 ± 2.7	150.1 ± 3.9	148.6 ± 4.9
HR peak (bpm)	NA	NA	162.0 ± 3.9	163.4 ± 4.2
RPE (AU)	NA	NA	4 ± 1.6	4.3 ± 1.4

Data are mean ± standard deviation; *n* = 12; NA = non-applicable because traditional photobiomodulation therapy (PBMT) and Placebo conditions did not perform any exercise/warm-up (i.e., participants remained seated at rest). WU, warm-up.

PRS showed no significant differences (*p* = 0.22) among conditions: WU + Placebo (8.5 ± 1.2 AU); WU + PBMT (8.4 ± 1.4 AU); placebo (9.2 ± 0.1 AU); PBMT (9.1 ± 0.8 AU). Similarly, no variations were observed in DOMS (*p* = 0.86): WU + Placebo (1.25 ± 1.28 AU); WU + PBMT (1.08 ± 0.99 AU); Placebo (1.33 ± 1.15 AU); PBMT (1.00 ± 0.73 AU). Baseline blood lactate concentrations exhibited no statistical differences (*p* = 0.46): WU + Placebo (1.9 ± 0.1 mmol. L^−1^); WU + PBMT (2.2 ± 0.3 mmol. L^−1^); Placebo (2.0 ± 0.5 mmol. L^−1^); PBMT (2.6 ± 0.7 mmol. L^−1^).

**Figure 2:**
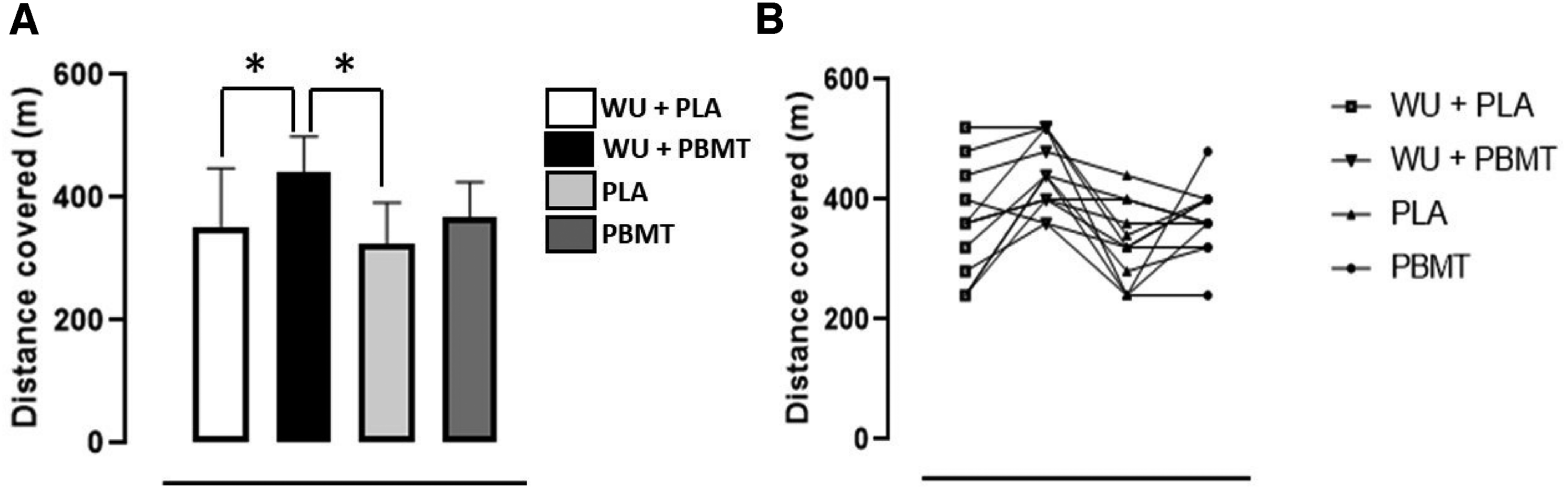
(**A**) Distance covered during the YoYo intermittent recovery test level 1 (YYIR1). *Different from WU + PBMT (*p* < 0.05). (**B**) Distance covered (m) individually. PLA, Placebo; PBMT, photobiomodulation therapy; WU, warm-up; *n* = 12.

Figure 2A illustrates the distance covered under all conditions. A significant difference (*p* = 0.02) emerged between WU + Placebo vs. WU + PBMT with an effect size (ES) of 1.0 (moderate), and WU + PBMT vs. Placebo with an ES of 1.8 (large). No differences were found in other comparisons: WU + Placebo vs. Placebo (*p* = 0.76) with ES of 0.3 (small); WU + Placebo vs. PBMT (*p* = 0.96) with ES of 0.1 (trivial); WU + PBMT vs. PBMT (*p* = 0.07) with ES of 1.2 (large). Finally, Placebo vs. PBMT (*p* = 0.48) with ES of 0.6 (small).

In the YYIR1 test, comparing individual performances under Placebo and PBMT conditions showed that 6 players improved with PBMT, 3 remained constant, and 3 experienced a decline. When evaluating WU + Placebo vs. WU + PBMT conditions, 10 players increased their distance covered with WU + PBMT, 1 maintained performance, and 1 demonstrated a decline. Figure 2B provides a visual representation of the individual distances covered in each condition.

Table 2 presents the responses of HR and RPE during YYIR1 and afterward across all conditions. No differences were found (*p* > 0.05).

**Table 2:** Heart rate (HR), rating of perceived exertion responses (RPE) and blood lactate responses during and after the YoYo intermittent test level 1 (YYIR1).

	PBMT	Placebo	WU + PBMT	WU + Placebo
HR min (bpm)	128.3 ± 13.0	120.3 ± 19.2	122.8 ± 12.6	124.7 ± 13.5
HR mean (bpm)	174.2 ± 7.8	171.1 ± 9.7	174.7 ± 3.4	181.7 ± 4.9
HR peak (bpm)	189.8 ± 4.1	188.8 ± 4.7	190.2 ± 3.9	189.9 ± 5.3
% HRmax	88.8%	87.2%	89.0%	92.6%
RPE (AU)	9.5 ± 0.6	9.4 ± 0.6	9.3 ± 0.8	9.5 ± 0.7
Post Lactate (mmol. L^−1^)	11.5 ± 3.0	12.3 ± 4.0	9.0 ± 2.6	12.4 ± 2.9
HR rec 1 min (bpm)	132.3 ± 11.2	136.4 ± 14.3	129.5 ± 13.5	134.7 ± 10.2
HR rec 2 min (bpm)	119.9 ± 13.2	122.3 ± 9.9	117 ± 10.8	120.7 ± 11.9
HR rec 3 min (bpm)	111.8 ± 14.3	113.2 ± 11.7	109.9 ± 12.1	110.8 ± 12.4

Data are mean ± standard deviation; *n* = 12; AU, arbitrary units; PBMT, photobiomodulation therapy; %HRmax, percentage values of maximum heart rate (220-age) over the HR mean; WU, warm-up; HR rec, HR recovery.

## 4 Discussion

The study aimed to assess the impact of PBMT, either alone or combined with a prior warm-up, on YYIR1 test performance and associated perceptual and physiological indicators. The primary discovery of this study was that post-standardized warm-up administration of PBMT significantly improved maximal intermittent running performance compared to conditions involving warm-up and placebo, as well as traditional PBMT without pre-warming up. Notably, this investigation represents the first exploration into the synergistic effects of muscle activation resulting from a specific warm-up (intermittent running) and the application of PBMT.

The baseline PRS, DOMS and the blood lactate concentration were not different (*p* > 0.05) in the four conditions, suggesting there was an equality from the physical condition and recovery among the four conditions.

The warm-up protocol, derived from a pilot study, aimed for modality-specific benefits, leveraging neural pathways and enhancing neuromuscular activation (9). The proposed warm-up achieved ∼76% of maximum HR. Prior research suggests such a warm-up enhances neural and enzymatic muscle fiber conduction velocity, possibly due to increased calcium release during fiber membrane depolarization (32).

We opted for the YYIR1 test due to its motor and physical similarities to the futsal game, involving actions like acceleration, deceleration, and changes of direction (17). Moreover, the test has proven effective in discerning players at different skill levels (33). Additionally, the YYIR1 has a significant aerobic contribution, and PBMT has been investigated as a potential ergogenic resource for activities with this characteristic (34). A previous study evaluated the effect of traditional (i.e., at rest) PBMT on YYIR1 in amateur futsal players and no performance optimization was found in the test (27). Nevertheless, the experimental design had more tests in the same session (before YYIR1) and there was no warm-up procedure before the PBMT/placebo.

In the current study, YYIR1 performance (distance covered) demonstrated a significant increase after WU + PBMT compared to only PBMT (∼74 m) and WU + Placebo (∼87 m). These results, not observed with the traditional use of PBMT (i.e., rest before PBMT) (27), indicate that preceding exercise can enhance the effectiveness of PBMT. Regarding ES, a moderate magnitude (1.0) was seen between WU + Placebo vs. WU + PBMT meaning practical relevance. This enhancement may have occurred because warm-up increases the turnover of ATP (9), which is a similar mechanism to the PBMT. During the PBMT application, the contact of light with Cox (complex IV) increases the flow of electrons in the electron transport chain, significantly expanding the amount of H^+^ ions, therefore increasing the availability of energy (ATP) (6). In summary, the combination of warm-up plus PBMT appears to enhance ATP availability in the muscles, resulting in improved performance, as evidenced by approximately two additional stages in the YYIR1.

No participant reported menstruating during the experiment, and the menstrual cycle phase is known not to affect YYIR1 performance (35). Overall, YYIR1's relative intensity (physiological and subjective) reached high/near-maximum levels across conditions (∼89.4% HRmax, blood lactate ∼11.3 mmol. L^−1^, RPE ∼9.4 AU). Despite no significant influence (*p* > 0.05) on these intensity variables among conditions, consistent with prior research (27), PBMT effects on these factors remain unclear in literature. Post-exercise lactate, for instance, may exhibit increased removal with PBMT after high-intensity exercise (3), contrasting studies showing no differences with similar high-intensity running (36). PBMT could enhance lactate removal through heightened mitochondrial capacity (3). Speculations regarding HR responses suggest PBMT may increase muscle oxygen extraction, potentially decreasing or maintaining HR values during submaximal intensities (36), yet no significant differences emerged among conditions, aligning with other studies (37).

Regarding the application parameters of the PBMT, we followed all the recommendations of a previous study: 200 J of energy dose for large muscle groups. In addition to the continuous mode, the application of 90 s per point, as well as the technique used in the application, which in this case was in direct contact with the skin with light pressure. Other parameters such as: when to irradiate, wavelength and power were also in accordance with guidelines (38).

Some limitations in this study were not assessing body temperature, microcirculation and muscle oxygenation in the muscles which received the PBMT/placebo. Readers should note that the study participants were amateur-level athletes; thus, findings may vary in elite-level performers. However, this is the first study that associates the use of a specific prior muscle activation with PBMT. Since other studies that investigated the ergogenic effect of PBMT used it passively or did not clearly present the muscle preparation prior to PBMT application in their experimental designs, the current study adds relevant information. Larger sample sizes in future studies are recommended to enhance result generalization and better understand PBMT effects, given inconclusive findings in existing literature with similar sample sizes and levels.

## 5 Conclusion

The integration of a standardized and tailored warm-up preceding PBMT application enhances high-intensity intermittent performance among amateur female futsal players, while demonstrating no impact on intensity variables such as heart rate (HR), lactate concentration, and perceived exertion.

## Data Availability

The raw data supporting the conclusions of this article will be made available by the authors, without undue reservation.
